# Elucidating the molecular markers and biological pathways associated with extrahepatic cholangiocarcinoma: a transcriptome sequencing study

**DOI:** 10.3389/fonc.2024.1417374

**Published:** 2024-09-17

**Authors:** Bin Zhao, Yanmei Gu, Daixiu Shi, Xiaokang Chen, Yumin Li

**Affiliations:** ^1^ Department of General Surgery, Lanzhou University Second Hospital, Lanzhou, China; ^2^ Department of the Second Clinical Medical College, Lanzhou University, Lanzhou, China; ^3^ Department of Nursing, Lanzhou University Second Hospital, Lanzhou, China

**Keywords:** extrahepatic cholangiocarcinoma, biomarkers, WGCNA, transcriptome sequencing, oncogene

## Abstract

**Background:**

Cholangiocarcinoma is a malignancy with high aggressiveness, and extrahepatic cholangiocarcinoma (ECCA) represents the predominant subtype. However, the molecular architecture and underlying pathogenic mechanisms of ECCA remain poorly understood. The objective of this study is to elucidate the molecular markers and biological pathways associated with ECCA.

**Methods:**

In order to identify the factors influencing ECCA, we conducted transcriptome sequencing on a cohort of 8 surgically resected ECCA specimens. To validate our findings, we integrated data from The Cancer Genome Atlas and Gene Expression Omnibus (GEO) databases using batch integration analysis. Finally, we confirmed our results using clinical samples.

**Results:**

The findings of this study reveal that through the analysis of sequencing data, we have successfully identified the genes that are differentially expressed and have a significant role in the development of ECCA. Utilizing the Weighted Gene Co-expression Network Analysis approach, we have integrated these identified gene modules with the GEO dataset, leading to the identification of four key genes (PTGDS, ITIH2, LSAMP, HBB) that are strongly associated with the progression-free survival of ECCA. We screened a key gene LSAMP from four genes using immunohistochemistry. The gene primarily participate in crucial biological processes such as the ECCA cell cycle and DNA replication. The qRT-PCR reaction and Western Blot conducted on the tissues provided confirmation of the expression levels of the gene, which exhibited consistency with the outcomes of our analysis.

**Conclusions:**

Our study has successfully identified potential biomarkers LSAMP for ECCA, which can serve as valuable tools for early detection and targeted therapeutic interventions in clinical settings.

## Introduction

Cholangiocarcinoma (CCA) is a prevalent gastrointestinal malignancy on a global scale, characterized by a notable recurrence rate and high mortality. The incidence of this malignancy has been steadily increasing over time (1). CCA can be classified into two main subtypes based on its anatomical location: intrahepatic cholangiocarcinoma (ICCA) and extrahepatic cholangiocarcinoma (ECCA). Among these subtypes, ECCA is the most frequently encountered. ECCA can be further categorized into two distinct subtypes: hilar cholangiocarcinoma and distal cholangiocarcinoma. The pathogenesis and genetic alterations of various types of cholangiocarcinoma may differ due to variations in tumor origin (2). Currently, treatment options for ECCA are limited, with surgical resection of the primary tumor being the primary approach. However, the 5-year survival rate for patients remains notably low, accompanied by a high risk of recurrence (3, 4). Molecular targeted therapy has not demonstrated a survival advantage for ECCA patients (5). Hence, it is imperative to explore molecular markers pertaining to the effectiveness of ECCA, comprehend its underlying biological mechanism, and devise innovative targets to enhance efficacy in patients. This approach holds potential advantages for personalized treatment of individuals with ECCA.

Tumor immune cell infiltration is currently a hot research topic, cholangiocarcinoma has its unique characteristics. A study found that neutrophils have the ability to present anti-tumor antigens (6, 7), another study found that tumor infiltrating B cells have become an important role in the immune response of cholangiocarcinoma and serve as predictive factors for immune therapy response, selective regulation of immune cell metabolism in cholangiocarcinoma, surpassing traditional thinking, heralds a new era of immunotherapy for cholangiocarcinoma (8).

The increasing utilization of technological advancements in the field of science has led to the widespread application of various technologies for the identification of cancer biomarkers (9). RNA-seq, a transcriptome analysis technique employing high-throughput sequencing methods, has been employed to unravel the intricate nature of eukaryotic transcriptomes. In a particular investigation, RNA-seq was employed to analyze clinical liver cancer tissues and their corresponding adjacent tissues, thereby identifying carcinogenic factors that influence the progression of liver cancer (10). Additionally, a separate study investigating factors influencing liver metastasis in colorectal cancer utilized second-generation sequencing to identify potential biomarkers (11). RNA-seq proves to be a potent instrument in the examination of cancer-related biological phenomena and the detection of biomarkers.

In this study, we conducted an analysis of transcriptional level expression in 8 ECCA and 8 matched paracancer tissues using second-generation sequencing. To identify significant genes associated with the disease, we integrated information from public databases and employed the Weighted Gene Co-expression Network Analysis (WGCNA) method. Subsequently, we established an ECCA prediction model based on the LASSO regression model, which included genes HBB, LSAMP, ITIH2, and PTGDS. The efficacy of the model was confirmed through the ROC curve, demonstrating its excellent prediction efficiency. Furthermore, we validated the expression of these genes in tissues. In order to elucidate the crucial pathways that impact the progression of ECCA, external analysis techniques such as Gene Ontology (GO) and Kyoto Encyclopedia of Genes and Genomes (KEGG) are employed. This investigation holds significant value in advancing the personalized therapeutic approaches for ECCA patients.

## Materials and methods

### Patients and samples

This study enrolled a total of 8 cases of ECCA, where tumor tissues of patients and their corresponding paracancer tissues were collected for second-generation sequencing. All patients were diagnosed with ECCA through pathological examination following surgery. The research protocol was approved by the review committee of the Second Hospital of Lanzhou University (approval no. 2020A-024) and conducted in accordance with the principles outlined in the Declaration of Helsinki.

### Transcriptome sequencing analysis

Following the extraction of RNA and preparation of the tissue samples, BGI Genomics offered technical assistance for the sequencing process. Utilizing the Illumina Hiseq platform, each sample generated an average data volume of 1.18G. The average rate of comparison amounted to 93.87%, with a total of 18905 genes identified. Subsequently, the data was filtered and quality control measures were implemented.

### Public database data download and collation

The extrahepatic cholangiocarcinoma dataset (GSE132305) was acquired from the GEO database, encompassing transcriptome data and grouping information. The dataset consisted of 182 instances of extrahepatic cholangiocarcinoma and 38 instances of para-cancerous tissue. Concurrently, the expression profile, clinical data, and survival information pertaining to cholangiocarcinoma from the TCGA database. Ultimately, a total of 36 cancerous tissues and 9 para-cancerous tissues were procured.

### Identification of differentially expressed genes

In our study, we employed the “DESeq2” package in R software to conduct an analysis of the expression levels of genes that exhibited differential expression in our cohort consisting of extrahepatic bile duct carcinoma and para-cancerous tissues. The criteria used for screening these differentially expressed genes (DEGs) were a |log2 Fold change| greater than 1 and a correct *P* value less than 0.05. The results of this analysis were visually represented through the use of volcanic maps, which showcased the identified DEGs.

### Functional enrichment analysis

The differential genes obtained from the analysis were subjected to enrichment analysis using the GO and KEGG databases. The Cluster profiler package was utilized to identify significantly enriched pathways within the GO and KEGG databases. A significant threshold of *P* < 0.05 was employed as the criterion for inclusion.

### WGCNA

In order to ascertain the crucial genes linked to the disease, we employed the WGCNA to construct co-expression gene templates. The ‘WGCNA’ package was utilized for analysis based on the data from DEGs. Subsequently, the co-expression analysis involved matching genes using the Pearson correlation matrix. The selection of the adjacency matrix weight parameter power of 14 was deemed necessary for achieving a scale-free topology fitting. Furthermore, a soft threshold power of 10 was set to identify significant modules. Finally, the calculation of module characteristic genes was performed for each module.

### Prognostic model construction

The disease related genes that were selected were included in the analysis for constructing the prognostic model. Using the survival information from the TCGA database, we conducted LASSO regression analysis using the “Survival” and “glmnet” packages in the R software. The risk score for each sample was calculated, and they were then categorized into high and low risk groups based on the median risk score. The ‘survival’ package was utilized to generate the survival curve, while the ‘timeROC’ package was employed to plot the time-dependent ROC curve, assessing the predictive efficiency of the model for 1-, 2-and 3-year survival.

### Immunohistochemical

Immunohistochemical staining was conducted on 53 pairs of ECCA and adjacent tissues from Lanzhou University Second Hospital. Sections were deparaffinized, rehydrated, and antigen-retrieved in EDTA Antigen Retrieval Solution (Beyotime Biotechnology) using microwave heating for 10 minutes. After blocking with 3% hydrogen peroxide (Maxim Biotechnologies) for 30 minutes and 10% normal goat serum (Maxim Biotechnologies) in PBS for 1 hour, sections were incubated overnight at 4°C with a primary antibody against PTGDS (1:100, cusabio, CSB-PA018969HA01HU),ITIH2(1:100, SAB 31939),LSAMP(1:50, Sangon Biotech, D123848),HBB(1:50, cusabio,CSB-PA010150LA01HU),. A biotinylated secondary antibody (Maxim Biotechnologies) was applied for 1 hour, followed by visualization with HRP-conjugated streptavidin (Maxim Biotechnologies) and DAB substrate ((Beyotime Biotechnology), and nuclei were counterstained with hematoxylin (Beyotime Biotechnology). Stained slides were examined and digitally imaged for quantitative analysis of UBE2M expression. Based on staining intensity, the scoring was classified into four levels: 0 (no staining), 1 (weak staining), 2 (moderate staining), and 3 (strong staining). The proportion of positive cells was categorized into the following levels: 0 (no positive cells), 1 (<10% positive cells), 2 (10-50% positive cells), 3 (51-80% positive cells), and 4 (>80% positive cells). The IHC score was calculated by multiplying the staining intensity by the proportion of positive cells. The relationship between PTGDS, ITIH2, LSAMP, HBB protein levels and survival outcomes were analyzed in these 53 patients using Kaplan-Meier survival curves, with differences between high and low expression groups assessed by the log-rank test.

### Quantitative real-time polymerase chain reaction

The identified key genes were validated at the mRNA level in the tissues through the utilization of qRT-PCR. Tissue RNA was extracted using the Trizol method, followed by reverse transcription of the total RNA into complementary DNA (cDNA) using a reverse transcription kit. Real-time PCR was subsequently conducted. The internal reference gene GAPDH was employed to establish the final threshold for each sample, allowing for the calculation of average error and standard error. The primer sequences relevant to this study can be found in **Table 1**.

**Table 1:** Primer sequence.

Gene	Sequence
LSAMP-F	AGAGTTCAGCCGGATCGGAA
LSAMP-R	CGTGCCTCGGTTAAAATCCAC
GAPDH-F	TGACATCAAGAAGGTGGTGAAGCAG
GAPDH-R	GTGTCGCTGTTGAAGTCAGAGGAG

### Western blot

Protein was extracted from 6 pairs of ECCA and adjacent tissues from Lanzhou University Second Hospital using RIPA buffer (solarbio life sciences) and quantified via the Bradford assay (Bio-Rad Laboratories). Proteins (30-50 µg) were separated on a 10% SDS-PAGE at 100 V for 1.5 hours and transferred to PVDF membranes (MilliporeSigma) at 250mA for 2 hours. Membranes were blocked with 5% non-fat milk and incubated with primary antibodies against LSAMP (1:1000, Assay Genie, CAB14248)) and Tubulin (1:5000, Proteintech Group,11224-1-AP) overnight at 4°C. After TBST washes, HRP-linked secondary antibodies (1:5000, Proteintech Group, B900120) were applied for 1 hour. Protein detection was performed using an ECL system (Amersham, GE Healthcare) and visualized with a gel documentation system (Epizyme Biotech), providing robust data on LSAMP levels.

## Result

### Identification of DEGs

**Figure 1:**
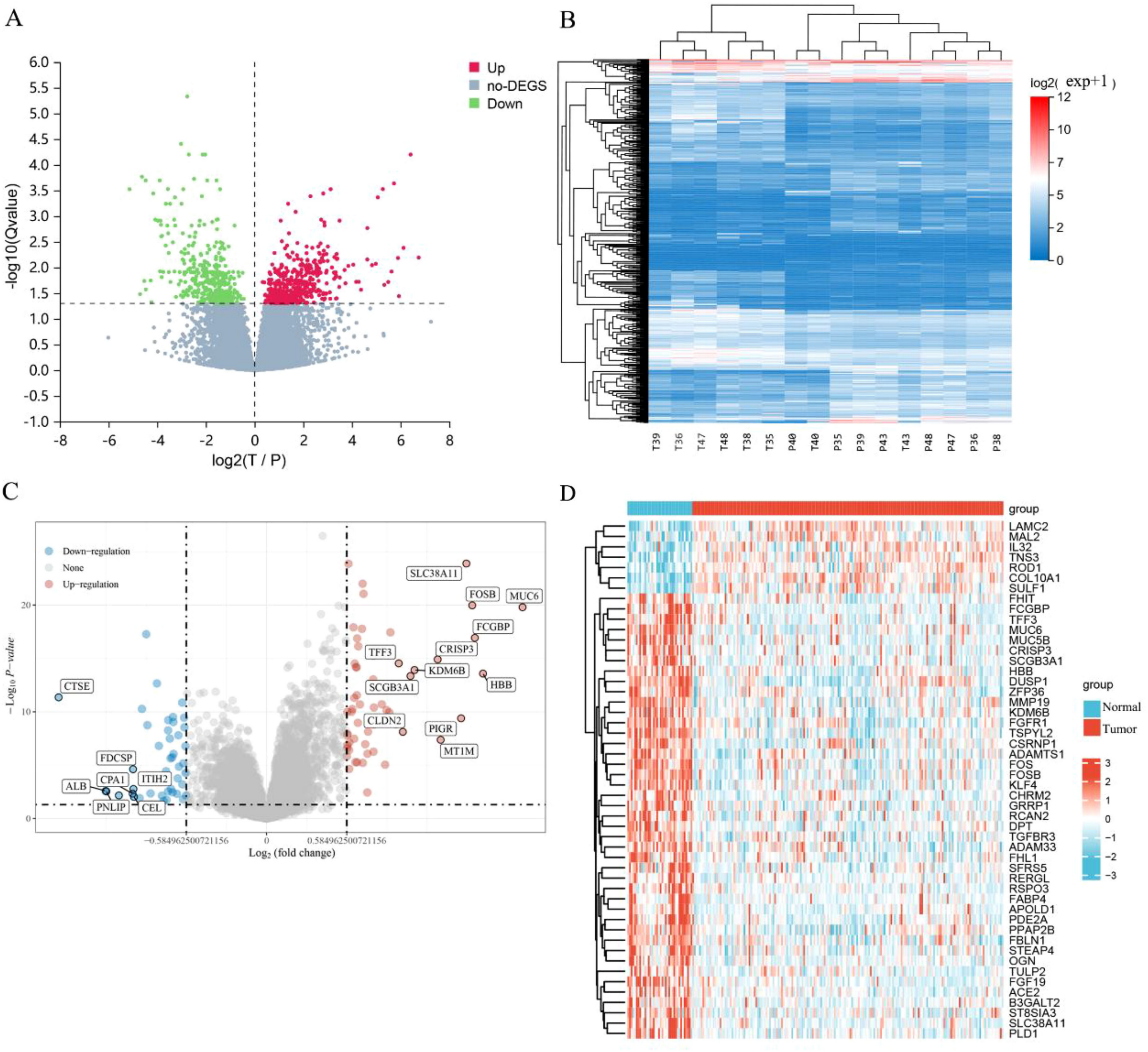
Identification of DEGs. **(A)** Differential gene volcano plot; **(B)** Differential gene heatmap; **(C)** Differential gene volcano plot of GEO; **(D)** Differential gene heatmap of GEO.

Following the completion of a differential expression analysis on a cohort of ECCA and paracancer tissues, a total of 729 genes exhibiting differential expression were identified (**Figures 1A, B**). Among these genes, 366 were found to be up-regulated, while 363 were down-regulated. Additionally, within the GEO data set, 100 differentially expressed genes were examined, with 57 showing up-regulation and 43 showing down-regulation (**Figures 1C, D**). We have listed the top 20 up-regulated and down-regulated DEGs in GEO (**Table 2**) and Transcriptome Sequencing (**Table 3**).

**Table 2:** The top 20 up-regulated and down-regulated DEGs in GEO.

Gene id	Log_2_FC	P.Value	Type
CTSE	-1.515817125	4.39475E-12	down
ITIH2	-1.172313295	0.002589415	down
HBB	-1.167847868	0.002853108	down
APOA2	-1.076388537	0.006850545	down
CPA1	-0.976069228	0.004624038	down
FDCSP	-0.97309119	2.39827E-05	down
ALB	-0.97027333	0.001688226	down
CEL	-0.966369077	0.009418242	down
CLPS	-0.925225768	0.012071965	down
FN1	-0.90838703	5.36808E-11	down
COL10A1	-0.877192396	5.3026E-18	down
POSTN	-0.868148554	1.78693E-09	down
APOC3	-0.845559915	0.004447826	down
SULF1	-0.798848284	2.19119E-13	down
HOPX	-0.784896324	1.54851E-07	down
CTRB2	-0.762638341	0.007190052	down
KNG1	-0.750569705	0.022486789	down
FAP	-0.732620388	3.66668E-05	down
RBP4	-0.728584346	0.018075976	down
CPB1	-0.717744144	0.002843687	down
MUC6	1.866085666	1.58811E-20	up
LSAMP	1.578145211	2.6261E-14	up
PTGDS	1.518396569	1.17527E-17	up
FOSB	1.499454346	1.064E-20	up
SLC38A11	1.45664561	1.28748E-24	up
PIGR	1.418233469	4.06903E-10	up
MT1M	1.269375287	4.22047E-08	up
CRISP3	1.247562129	1.23067E-15	up
KDM6B	1.078868002	1.21515E-14	up
SCGB3A1	1.04915524	4.43099E-14	up
CLDN2	0.993490185	7.57704E-09	up
TFF3	0.963754844	2.88606E-15	up
SPARCL1	0.942882468	1.48517E-09	up
ADAMTS1	0.903098913	3.60496E-18	up
C11ORF96	0.895157252	1.12667E-10	up
CXCL2	0.875428473	6.90041E-11	up
TFF2	0.864346726	9.05063E-06	up
FABP4	0.859682649	2.95702E-13	up
PTGDS	0.840212849	1.97509E-11	up
EGR1	0.782932178	3.93232E-11	up

**Table 3:** The top 20 up-regulated and down-regulated DEGs in Transcriptome Sequencing.

Gene id	Log_2_FC	P.Value	Type
KRT6A	6.743584382	0.006414124	up
ANXA8L1	6.41024893	0.0000632	up
UPK2	6.123274756	0.004154234	up
IGFL1	5.932382874	0.036083864	up
PGLYRP4	5.894737873	0.006541637	up
MUC16	5.729670106	0.00023	up
KLK5	5.627670517	0.011978517	up
POTEE	5.49333191	0.019134075	up
STYXL2	5.330315194	0.021754412	up
FAM83A	5.272903902	0.000297	up
KLK6	5.063388777	0.00043	up
IBSP	4.984081516	0.008462593	up
PADI3	4.825820873	0.009009625	up
KLK13	4.635229557	0.001707082	up
ITIH6	4.632969954	0.006965831	up
GPR87	4.374522164	0.027153775	up
CALHM3	4.28242351	0.018997492	up
LIPF	4.176612051	0.01898084	up
SAA4	4.058294576	0.008828529	up
FOXJ1	3.851500226	0.009343895	up
PAH	-5.135339922	0.000299	down
UCMA	-4.70556523	0.032987108	down
CPB2	-4.627017005	0.00017	down
SCGB3A1	-4.528784204	0.01827537	down
UGT2B15	-4.475026438	0.000202	down
LOC101929773	-4.4679697	0.026794447	down
OSTN	-4.265229107	0.017904372	down
CYP1A1	-4.234891511	0.047454727	down
CRISP3	-4.165137665	0.000362	down
CHRM2	-4.075813871	0.001168454	down
HBA1	-3.969200406	0.001225254	down
TEX26	-3.897174144	0.000202	down
NPBWR1	-3.88206847	0.011881354	down
C8A	-3.866896901	0.001222306	down
HBA2	-3.844315517	0.001475643	down
SERPINA6	-3.833389895	0.002478923	down
HBG2	-3.813267955	0.015384848	down
HBB	-3.801632715	0.002421776	down
DCAF12L1	-3.753432386	0.012184392	down
GC	-3.649902101	0.003736276	down

### GO and KEGG analysis

**Figure 2:**
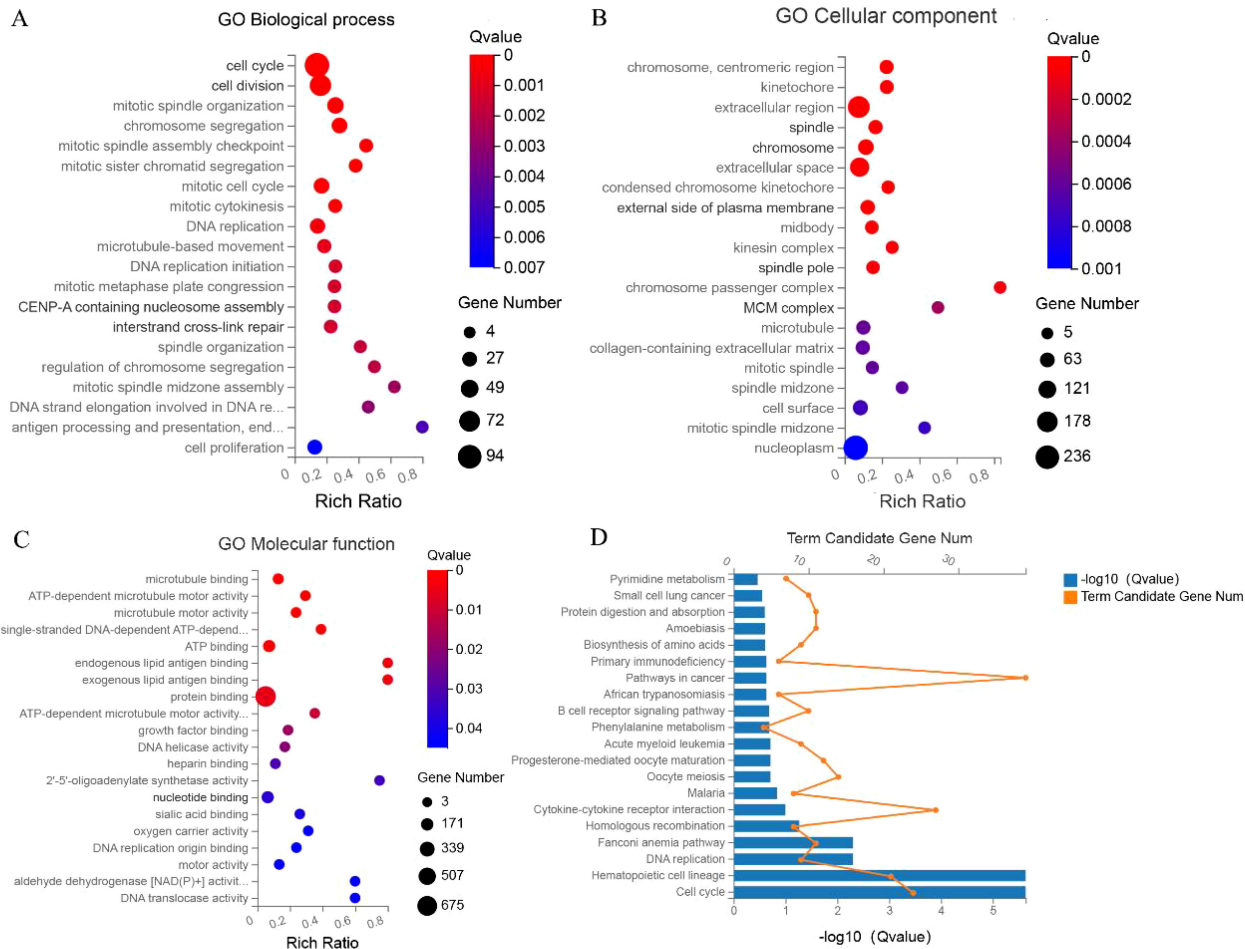
GO and KEGG analysis **(A–C)** GO analysis; **(D)** KEGG.

The GO functional enrichment analysis encompasses three ontologies, namely molecular functions, cell components, and biological processes, which collectively provide a comprehensive depiction of gene properties within an organism. In this study, we performed GO enrichment analysis on the selected DEGs, revealing their predominant involvement in cell cycle regulation, mitotic processes, microtubule dynamics, and DNA replication, specifically within the context of ECCA (**Figures 2A–C**). Furthermore, we conducted a detailed examination of the functional roles played by the aforementioned differentially expressed genes in ECCA. We conducted a comprehensive analysis of the functions of the aforementioned differentially expressed genes in ECCA. Our findings indicate that these genes play significant roles in the regulation of cancer signaling pathways, including but not limited to cell cycle, DNA replication, pyrimidine metabolism, and phenylalanine metabolism (**Figure 2D**).

### Screen for modules and genes associated with the disease

**Figure 3:**
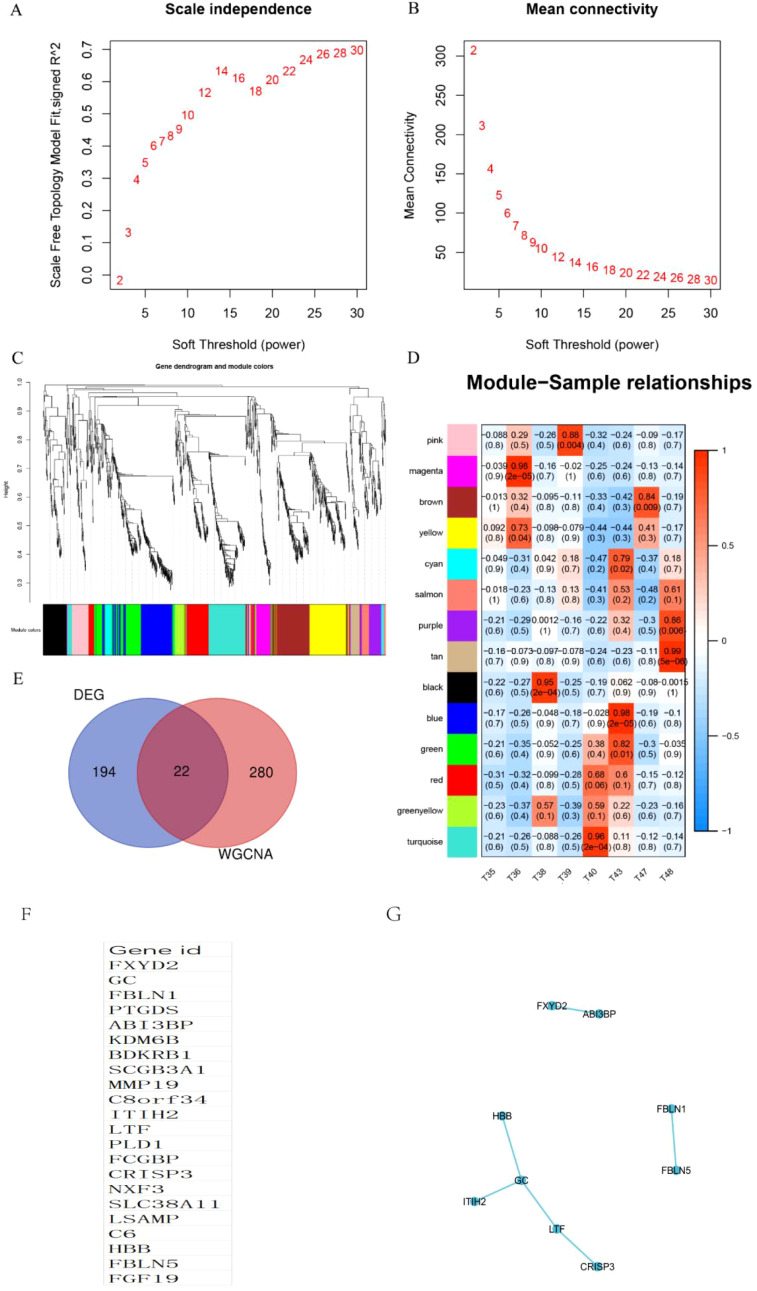
Identification of WGCNA modules. **(A, B)** Scale independence and mean connectivity were used for soft threshold selection in WGCNA. **(C)** Gene dendrogram showing co-expression modules identified by WGCNA. **(D)** Correlation analysis result between WGCNA module and sample subtype; **(E)** Venn chart of hub genes; **(F)** List of 22 hub genes; **(G)** PPI analyses of 22 hub genes.

Using the differential genes identified within this cohort, a co-expression network was constructed utilizing the WGCNA approach. The soft threshold capability, β=3, was selected for this purpose (**Figures 3A, B**). A hierarchical clustering tree was then generated through dynamic hybrid cutting, wherein each leaf on the tree corresponds to a gene. The genes exhibiting similar expression patterns are situated in close proximity, thereby forming a branch on the tree and representing a gene module (**Figure 3C**). Ultimately, three distinct modules were established, with the blue and brown modules displaying a strong correlation with ECCA, surpassing a correlation coefficient of 0.5. The blue module was specifically identified as the hub module (**Figure 3D**). To identify disease-related hub genes, we conducted a cross-linkage between the hub module in WGCNA and the DEGs identified by GEO. Ultimately, we successfully identified a total of 22 hub genes (**Figure 3E**).We have listed 22 hub genes (**Figure 3F**) and PPI analyses (**Figure 3G**). The KEGG pathways analyses of 22 hub genes have been listed (**Table 4**).

**Table 4:** The KEGG pathways analyses of 22 hub genes.

ONTOLOGY	ID	Description	pvalue	geneID
BP	GO:0030198	extracellular matrix organization	0.000274779	FBLN1/ABI3BP/MMP19/FBLN5
BP	GO:0043062	extracellular structure organization	0.000278204	FBLN1/ABI3BP/MMP19/FBLN5
BP	GO:0045229	external encapsulating structure organization	0.000285147	FBLN1/ABI3BP/MMP19/FBLN5
CC	GO:0070820	tertiary granule	2.53378E-05	LTF/PLD1/CRISP3/HBB
CC	GO:1904724	tertiary granule lumen	2.68544E-05	LTF/CRISP3/HBB/PTGDS
CC	GO:0071682	endocytic vesicle lumen	0.000273055	LTF/HBB
CC	GO:0072562	blood microparticle	0.000498305	GC/ITIH2/HBB
CC	GO:0042581	specific granule	0.000637886	LTF/PLD1/CRISP3/LSAMP
CC	GO:0062023	collagen-containing extracellular matrix	0.001009065	FBLN1/ABI3BP/ITIH2/FBLN5
CC	GO:0035580	specific granule lumen	0.00199015	LTF/CRISP3
CC	GO:0030139	endocytic vesicle	0.005551815	LTF/PLD1/HBB
CC	GO:0031838	haptoglobin-hemoglobin complex	0.011729327	HBB
CC	GO:0005833	hemoglobin complex	0.012789108	HBB
CC	GO:0005890	sodium:potassium-exchanging ATPase complex	0.012789108	FXYD2
CC	GO:0044666	MLL3/4 complex	0.014905423	KDM6B
CC	GO:0090533	cation-transporting ATPase complex	0.019125098	FXYD2
CC	GO:0098533	ATPase dependent transmembrane transport complex	0.025422347	FXYD2
CC	GO:0046930	pore complex	0.027512861	C6
MF	GO:0005201	extracellular matrix structural constituent	0.000941702	FBLN1/ABI3BP/FBLN5
MF	GO:0061134	peptidase regulator activity	0.002167619	FBLN1/ITIH2/LTF

### Identification of gene sets associated with survival

**Figure 4:**
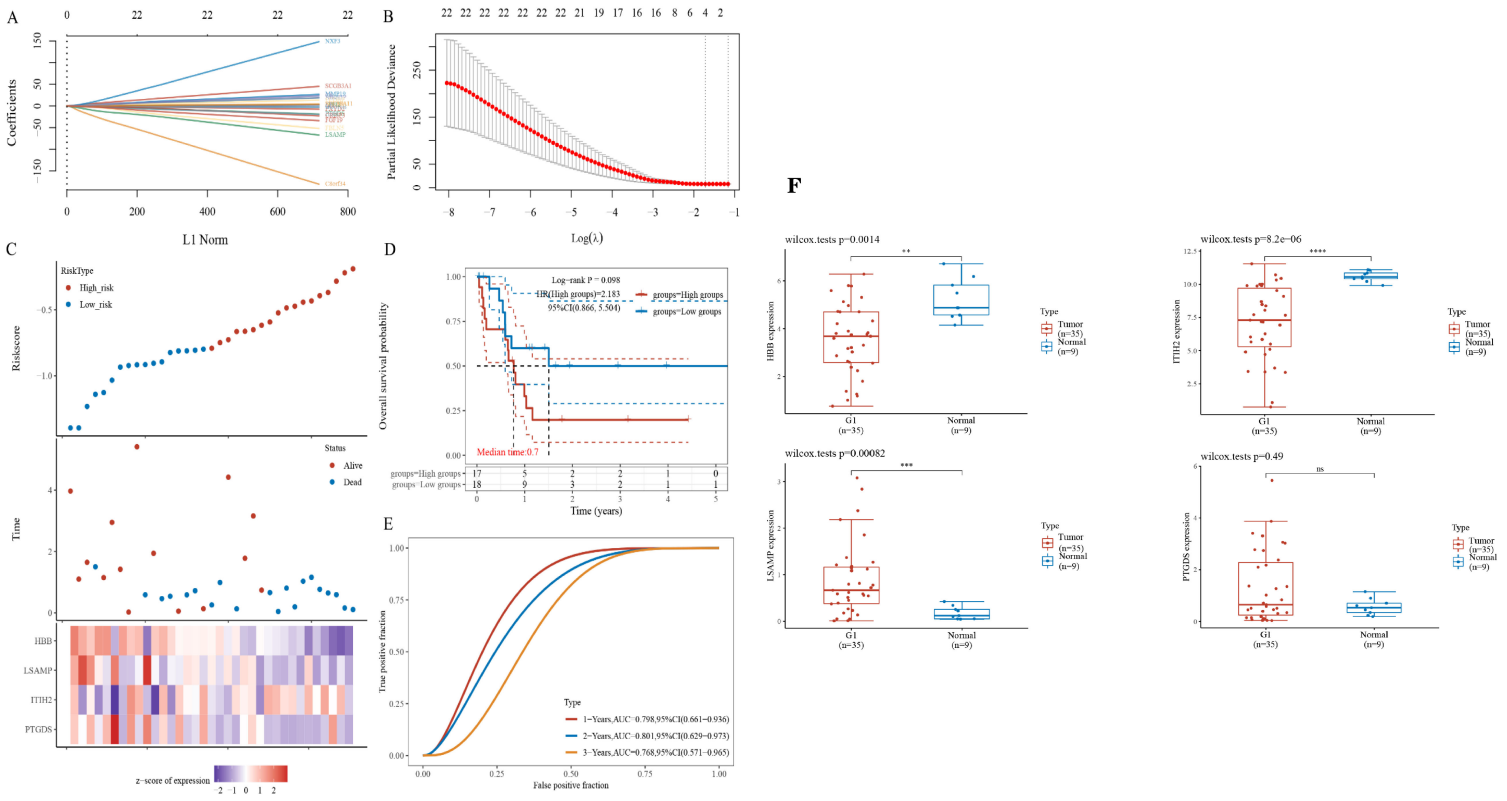
Identification of gene sets associated with survival. **(A, B)** LASSO regression of prognostic genes. **(C)** The gene expression heatmap between high- and low-riskscore groups. **(D)** Survival curves between high- and low-riskscore groups; **(E)** ROC curves for 1-, 2- and 3- year survival. **(F)** Relative expression of hub genes based on the TCGA database. ** P<0.01, *** P<0.001 , **** P<0.0001, ns=no significant P>0.05.

To evaluate the potential of the hub gene as a biomarker, we employed LASSO regression to construct a model. Given that the TCGA cholangiocarcinoma dataset encompasses progression-free survival data, we utilized this dataset to develop prognostic models. The risk model, derived from LASSO regression, incorporates the following risk scoring formula: Riskscore = (-0.046) * PTGDS + (0.0043) * ITIH2 + (-0.1817) * LSAMP + (-0.1574) * HBB (**Figures 4A, B**). The model encompasses a total of four candidate genes. Subsequently, we categorized patients into high-risk and low-risk groups based on the median risk score. Patients were subsequently categorized into high-risk and low-risk groups according to the median risk score. The figure presented demonstrates a positive correlation between the risk score and the number of patients in a deceased state (**Figure 4C**). The survival curve analysis revealed that patients in the high-risk group exhibited significantly lower PFS compared to those in the low-risk group (**Figure 4D**). However, this difference did not reach statistical significance. Subsequently, receiver operating characteristic (ROC) curves were employed to assess the model’s predictive performance (**Figure 4E**). The findings indicated that the model effectively predicted 1-year, 2-year, and 3-year PFS in patients with ECCA.We integrated the expression results of PTGDS, ITIH2, LSAMP, and HBB from the TCGA database (**Figure 4F**).

### Verifying the expression of hub genes

**Figure 5:**
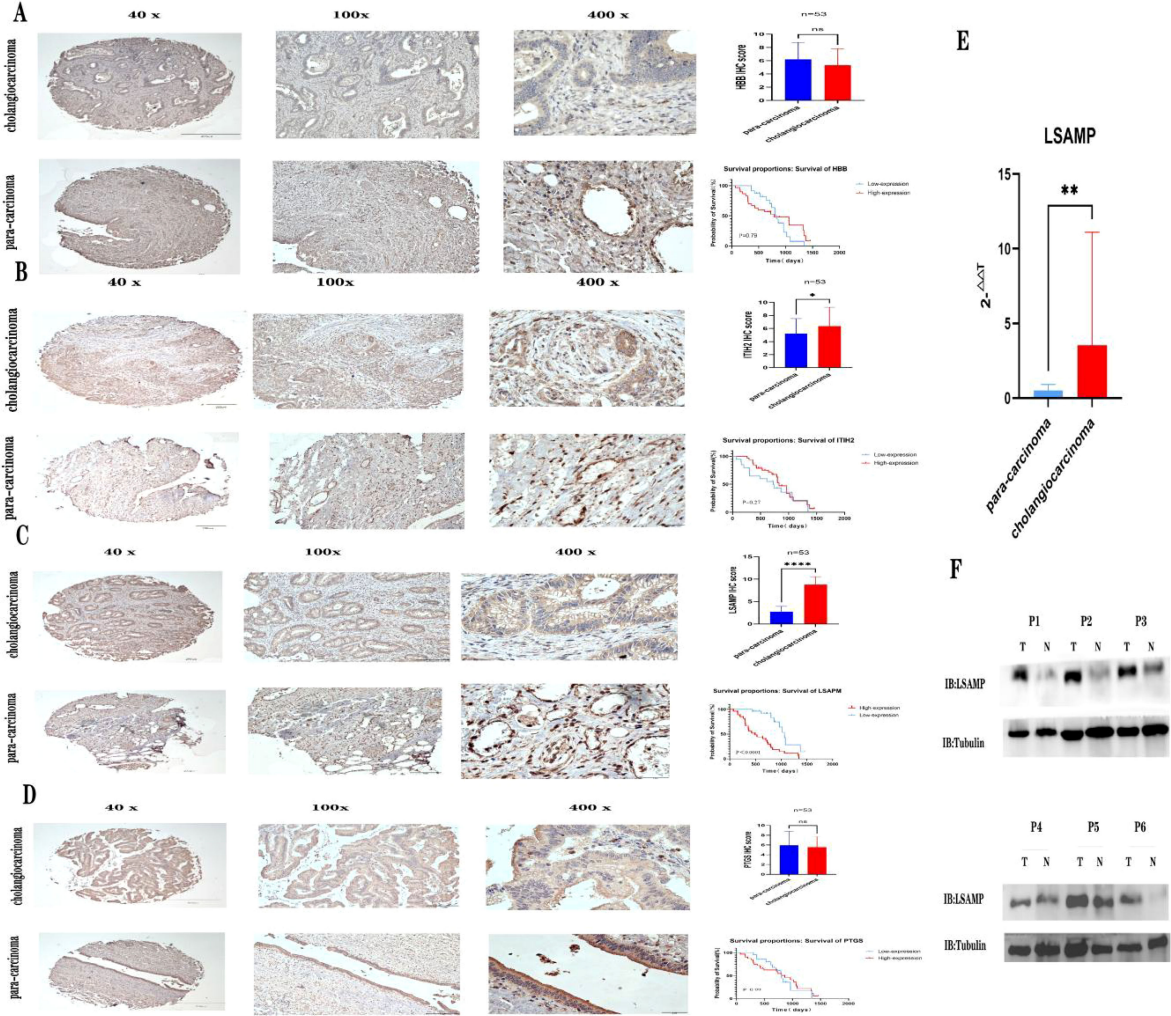
Verifying the expression of hub genes. **(A)** Expression of HBB immunohistochemistry and patient prognosis; **(B)** Expression of ITIH2 immunohistochemistry and patient prognosis; **(C)** Expression of LSAMP immunohistochemistry and patient prognosis; **(D)** Expression of PTGS immunohistochemistry and patient prognosis; **(E)** LSAMP mRNA expression levels from quantitative RT-PCR in 15 pairs of cancer tissues and adjacent tissues. **(F)** Western blot images showing LSAPM protein levels in six pairs of cancer tissues and adjacent tissues. * P<0.05 , ** P<0.01, **** P<0.0001, ns=no significant P>0.05.

**Figure 6:**
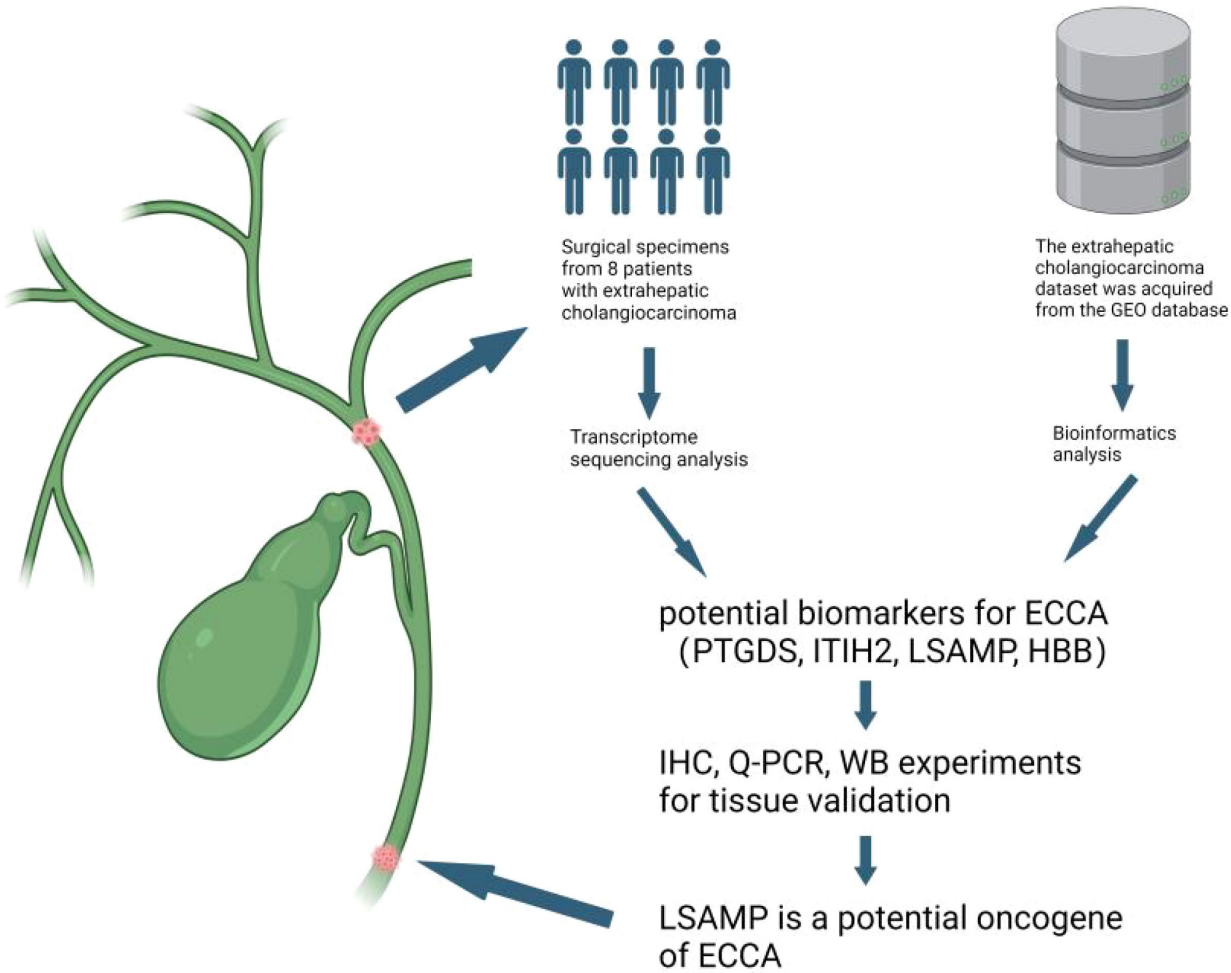
Discovery of potential biomarkers LSAPM for ECCA. We conducted transcriptome sequencing on a cohort of 8 surgically resected ECCA specimens,then we integrated data from The Cancer Genome Atlas and Gene Expression Omnibus (GEO) databases using batch integration analysis. Finally, we confirmed our results using clinical samples.Figure created with BioRender.com.

In order to confirm the expression of the aforementioned genes, Immunohistochemical was conducted on a total of 53 pairs of ECCA tissues and adjacent tissues. Comparative analysis revealed that PTGDS, and HBB exhibited lower expression levels in tumor tissues compared to normal tissues (**Figures 5A**, **D**, P>0.05).Comparative analysis revealed that ITIH2, and LSAMP exhibited higher expression levels in tumor tissues compared to normal tissues (**Figures 5B**, **C**, P<0.05), high expression of ITIH2 in cancer tissue is not associated with patient prognosis (**Figure 5B**, P>0.05), high expression of LSAMP in cancer tissue is associated with patient prognosis (**Figure 5C**, P<0.05). In addition, we conducted qRT PCR validation on cancer tissues and adjacent tissues of 15 cholangiocarcinoma patients and found that LSAMP expression was significantly higher in cancer tissues than in adjacent tissues (**Figure 5E**, P<0.05). We conducted WB validation on cancer tissues and adjacent tissues of 6 cholangiocarcinoma patients and found that the expression of LSAMP in cancer tissues was significantly higher than that in adjacent tissues (**Figure 5F**). Therefore, LSAMP is an important oncogene for cholangiocarcinoma (**Figure 6**).

## Discussion

Cholangiocarcinoma, a profoundly malignant tumor affecting the digestive system, predominantly manifests as ECCA. The availability of efficacious treatment modalities is constrained, resulting in a persistently low five-year survival rate (5, 12). Currently, the molecular underpinnings of ECCA remain elusive, necessitating the expedited exploration of novel biomarkers to revolutionize the diagnosis and therapeutic approaches for ECCA.

This study utilized a combination of second-generation sequencing and multi-database analysis to successfully identify a gene set that is significantly associated with the occurrence and progression of ECCA. The identified genes, namely PTGDS, ITIH2, LSAMP, and HBB, have been found to be closely linked to ECCA. Specifically, PTGDS is a prominently expressed n-glycosylated protein in various tissues, which exerts control over numerous biological processes and plays a crucial role in prostaglandin metabolism and lipid transportation. PTGDS has been demonstrated to participate in inflammatory processes within organisms (13). Increasing evidence suggests that PTGDS also plays a role in cancer processes. PTGDS has been observed to be upregulated in patients, such as those with diffuse large B-cell lymphoma, and is correlated with unfavorable outcomes. Through its interaction with MYH9, PTGDS modulates the Wnt signaling pathway and exerts a pro-tumor effect (14). Additionally, PTGDS is involved in the regulation of migration and invasion in testicular cancer cells (15). A diminished expression of PTGDS was observed in ECCA, indicating its potential role as a protective factor against normal tissues.

Inter-alpha-trypsin inhibitor heavy chain 2 (ITIH2), a constituent of the ITI protein family, plays a crucial role in stabilizing the extracellular matrix and impeding tumor metastasis. Numerous investigations have substantiated ITIH2 as a significant molecular indicator for cancer (16–19). Nevertheless, the precise mechanism through which ITIH2 operates in cancer remains ambiguous. Cancer progression has been linked to the involvement of Beta-globin (HBB) in various pathways. One such pathway involves the inhibition of intracellular reactive oxygen species by HBB, which aids in protecting cancer cells from entering the circulation and facilitates cancer metastasis (20). LSAMP has been recognized as a tumor suppressor in some cancers (21, 22). In the context of osteosarcoma, LSAMP has been frequently observed to be absent, which is strongly associated with tumor advancement. The potential mechanism by which LSAMP exerts its effect may involve the indirect up-regulation of HES1 and CTAG2, as suggested by previous research (23). A study suggests that high expression of LSAPM in ovarian cancer can serve as a specific target for ovarian cancer immunotherapy (24).Our study has found that LSAMP is highly expressed in extrahepatic cholangiocarcinoma tissues and it is highly correlated with patient prognosis.

## Conclusion

In summary, a thorough examination of the transcriptome of ECCA was performed, resulting in the discovery of novel molecular indicators. This study establishes a reference point for the advancement of targeted therapeutic approaches in ECCA through molecular analysis. We have found that LSAMP is a key oncogene in cholangiocarcinoma, providing a theoretical basis for future treatment of cholangiocarcinoma.

## Data Availability

The original data presented in the study are included in the article/supplementary material, further inquiries can be directed to the corresponding author.
